# Editorial: Multimodal Tracking of Functional Data in Parkinson's Disease and Related Disorders–Speech and Language Neuromotor and Cognitive Assessment

**DOI:** 10.3389/fnhum.2021.750075

**Published:** 2021-09-17

**Authors:** Athanasios Tsanas, Jiri Mekyska, Réjean Plamondon, Pedro Gomez-Vilda

**Affiliations:** ^1^Edinburgh Medical School, Usher Institute, University of Edinburgh, Edinburgh, United Kingdom; ^2^Faculty of Electrical Engineering and Communication, Department of Telecommunications, Brno University of Technology, Brno, Czechia; ^3^Polytechnique Montréal, Montreal, QC, Canada; ^4^Center for Biomedical Technology, Universidad Politécnica de Madrid, Madrid, Spain

**Keywords:** Parkinson's disease, neurodegenerative disorders, multimodal tracking, speech and language assessment, neuromotor symptoms

Parkinson's Disease (PD) and related disorders are a growing concern worldwide due to their chronic nature, with enormous implications for national health systems. Indicatively, there were ~2.5 million People diagnosed with PD (PwP) in 1990, and 6.1 million PwP in 2016 (GBD 2016 Parkinson's Disease Collaborators, 2018), with prevalence and incidence rates expected to rise further as the population grows older. Patients with these chronic disorders require treatment, continual palliative attention, rehabilitation, and caregiving. An *ad-hoc* functional monitoring allowing for a detailed data collection, dedicated to attempt a representation of the symptoms in patients' everyday life, is needed to provide actionable insights into symptom trajectories and disease progression. Notably, wearable sensors have been used so far in PD, detecting tremor and gait abnormalities for example. Functional Monitoring can be performed via Multimodal Tracking using Machine Learning Methods. Multimodal Tracking relies on the use of simple non-invasive methods applied to patients who are home-bound (thus enabling e.g., de-centralized clinical trials) or are being treated in care associations. Tracking aims to collect data at different levels, capitalizing on the diverse toolkit of sensors that is available and becoming increasingly affordable. Machine Learning Methods process large amounts of implicitly related correlates from axial motor symptoms, as limb movement, for instance in handwriting with speech-related symptoms affecting respiration, phonation, articulation, and fluency. Different digital endpoints have been proposed in the last years to monitor PD and related disorders' patients. There has been an increased focus on speech signal analysis, handwriting evaluation, gait, and body movement assessment, and object handling, as well as cognitive testing. Multimodal Tracking related to Language and Speech evaluation may, for example, refer to a combination of Surface Face and Limb Electromyography (sFLEMG), Electroencephalography from speech-production related brain areas (EEG), 3D Face and Limb Accelerometry (3DFLAcc), Facial Image Recognition and Monitoring (FIRM), Speech Recordings (SR), Electropalatography (EPG), amongst others.

The purpose of the Research Topic was to present a detailed view of the current state of the art and provide a forum to highlight current attempts of research work in this field, focusing on speech production, language processing, writing and motor abilities, that are notably and evidently affected in the progression of the disorders, in particular PD.

Among the received submissions, 4 were ultimately accepted for publication in this special issue. We summarize in brief the contributions of those papers below, and encourage interested readers to study the corresponding papers for further in-depth information.

Chen et al. tackled the challenging problem where some PwP may freeze during walking, known as Freezing of Gait (FoG). They reported findings on 53 early- to mid-stage PD, where participants were requested to perform six tasks of walking adaptability following a standardized assessment program (C-Gait). They presented evidence that carefully observed gait assessment though C-Gait could provide further additional information compared to traditional PD walking tests and lead to more personalized monitoring FoG insights.

Letanneux et al. focused on the Non-Motor Symptoms (NMS) in PD, and specifically on a lexical decision task to assess whether (1) the mental lexicon is impaired in PwP and (2) performance is affected by bradykinesia. The reported on 34 PwP (almost equally split between “on” and “off” medication) who did not have dementia symptoms and 19 age matched controls, performing a lexical decision task and a motor task. They found that motor task performance was not affected in PwP compared to matched controls, however unmedicated PwP where slower than controls to react to stimuli.

Lopez-de-Ipina et al. reported on Essential Tremor (ET) integrating handwriting and neuroimaging analysis. They analyzed data from 19 people diagnosed with ET who underwent MRI assessment and completed an automated Archimedes' spiral task (considered the “the gold standard” reference test for the clinical diagnosis of ET). They demonstrated how the analysis of fine motor skills, as measured by an automated Archimedes' spiral task, is correlated with neuroimaging biomarkers for ET. They conclude that their findings confirm the concordance of findings between the clinical examination and neuroimaging results, even in low amplitude tremors.

Hidalgo-De la Guía et al. conducted an exploratory study to investigate whether the neuromotor deficits in children with the Smith–Magenis Syndrome (SMS) adversely affect phonation as compared to typically developing children without neuromotor deficits. They compared the phonatory performance of 12 children with SMS aged 5–12 years old with an age- and gender-matched cohort of 12 children, using established acoustic measures which characterized sustained vowel /a/ phonations. They provided evidence that the neuromotor deficits that characterize children with SMS may adversely affect laryngeal biomechanics and thus vocal quality.

## Conclusion

The articles in this topic highlight both the complexity and the diversity of approaches in monitoring and assessing the pathophysiology of different chronic neurological disorders and provide new insights into potential approaches toward assessment and management. Although these four studies report on a small number of participants and findings would need to be validated to larger cohorts, we believe they make some very useful contributions to the research literature.

## Author Contributions

AT and PG-V wrote the first draft of the editorial. All co-editors of this special issue provided comments and approved the submitted version. All authors contributed to the article and approved the submitted version.

## Conflict of Interest

The authors declare that the research was conducted in the absence of any commercial or financial relationships that could be construed as a potential conflict of interest.

## Publisher's Note

All claims expressed in this article are solely those of the authors and do not necessarily represent those of their affiliated organizations, or those of the publisher, the editors and the reviewers. Any product that may be evaluated in this article, or claim that may be made by its manufacturer, is not guaranteed or endorsed by the publisher.
